# Assembly of mRNA-Protein Complexes for Directional mRNA Transport in Eukaryotes - An Overview

**DOI:** 10.2174/138920312801619493

**Published:** 2012-06

**Authors:** Ralf-Peter Jansen, Dierk Niessing

**Affiliations:** 1 Interfaculty Institute for Biochemistry, University of Tübingen, Tübingen, Germany; 2 Institute of Structural Biology, Helmholtz Zentrum München–German Research Center for Environmental Health, München, Germany; 3 Gene Center and Department of Biochemistry, Ludwig-Maximilians-University, München, Germany

**Keywords:** *ASH1* mRNA, cytoskeleton, *K10* mRNA, motor proteins, mRNA localization, *oskar* mRNA, RNA-binding proteins, translational control.

## Abstract

At all steps from transcription to translation, RNA-binding proteins play important roles in determining mRNA function. Initially it was believed that for the vast majority of transcripts the role of RNA-binding proteins is limited to general functions such as splicing and translation. However, work from recent years showed that members of this class of proteins also recognize several mRNAs via cis-acting elements for their incorporation into large motor-containing particles. These particles are transported to distant subcellular sites, where they become subsequently translated. This process, called mRNA localization, occurs along microtubules or actin filaments, and involves kinesins, dyneins, as well as myosins. Although mRNA localization has been detected in a large number of organisms from fungi to humans, the underlying molecular machineries are not well understood. In this review we will outline general principles of mRNA localization and highlight three examples, for which a comparably large body of information is available. The first example is She2p/She3p-dependent localization of *ASH1* mRNA in budding yeast. It is particularly well suited to highlight the interdependence between different steps of mRNA localization. The second example is Staufen-dependent localization of *oskar* mRNA in the *Drosophila* embryo, for which the importance of nuclear events for cytoplasmic localization and translational control has been clearly demonstrated. The third example summarizes Egalitarian/Bicaudal D-dependent mRNA transport events in the oocyte and embryo of *Drosophila.* We will highlight general themes and differences, point to similarities in other model systems, and raise open questions that might be answered in the coming years.

## INTRODUCTION

Transcriptional control of gene expression was long considered as the main mechanism to regulate cellular functions. The identification of large networks of posttranscriptional modifications challenged this view. They also demonstrated the great advantage of rapid response to external stimuli. More recently, it emerged that also translational control contributes to a wide range of biological processes. It shares with posttranslational modifications the advantage of rapid response, but also offers the great benefit of a regulated amplification of the genetic information from a single transcript into dozens to hundreds of protein molecules. 

Translation of mRNAs can be regulated in time (temporal control), but also by controlling the location of protein synthesis (spatial control). Often these two properties are combined to achieve a tight control over protein synthesis. Subcellular localization of mRNAs is used to generate asymmetric accumulation of proteins and thus cellular asymmetry. A genome-wide assessment of the intracellular distribution of mRNAs in embryos of *Drosophila melanogaster* yielded the surprising insight that about 70 % of the expressed transcripts are subcellularly localized [1]. Although *Drosophila* embryogenesis might be an extreme case, it demonstrates the importance of this mechanism of gene regulation. Indeed, data from the last 20 years showed that mRNA localization is very common in eukaryotes [2]. It has been studied in fungi [3], invertebrates like *Drosophila melanogaster* and *Caenorhabditis elegans* [4], and vertebrates such as *Xenopus laevis*, mice and human cell lines [5].

**Fig. (1):**
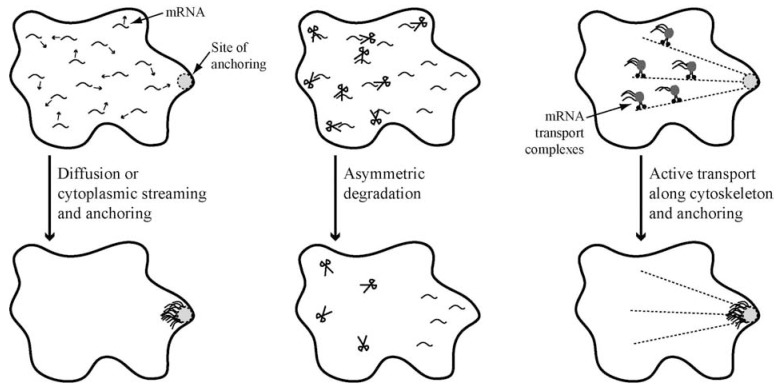
**Three mechanisms for asymmetric subcellular mRNA distribution. Left**: Diffusion and cytoplasmic streaming bring mRNAs to particular sites of a cell and allow for their transcript-specific subcellular anchoring. **Middle**: Asymmetric mRNA degradation results in local depletion of transcripts and cellular asymmetry of a particular mRNA. RNase activity is depicted as scissors. **Right**: Directional mRNA transport by motor-protein containing particles along microtubules or actin filaments is the most efficient way to ensure strictly localized translation of particular transcripts. Combinations of these three mechanisms are possible.

An asymmetric distribution of a subset of transcripts can be achieved by different mechanisms. One possibility is that transcripts simply diffuse through the cytoplasm or reach a certain subcellular site by cytoplasmic streaming, where they become anchored and subsequently translated (Fig. **1**, left) [6]. Local accumulation and translation of transcripts can also be achieved by their asymmetric degradation in certain regions of a cell, leaving only mRNAs in a particular region intact (Fig. **1**, middle) [6, 7]. The third and most studied mechanism of mRNA localization involves the directional transport of transcripts along the cytoskeleton network (Fig. **1**, right). 

**Fig. (2):**
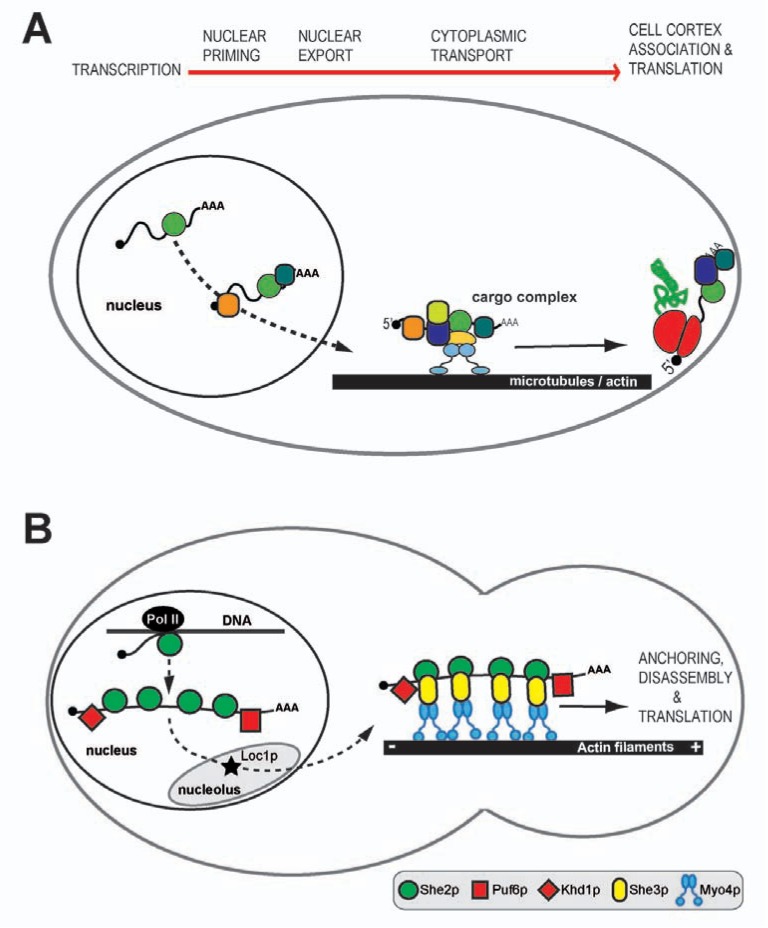
**Motor-dependent mRNA localization. A:** General model for mRNA localization. On top is an outline of the main stages of this process. Nuclear priming and cell cortex association/anchoring are not necessarily present in all mRNA localization events. **B:** *ASH1* mRNA localization during mitosis of the budding yeast *Saccharomyces cerevisisae*. This active transport event is mediated by a type V myosin motor towards the plus-ends of actin filaments. To date, it constitutes the most comprehensively understood mRNA localization event.

In this last scenario, mRNAs are recognized by dedicated RNA-binding proteins and transported as part of motor-protein containing messenger ribonucleoprotein particles (mRNPs) to subcellular sites most often in the periphery of a cell [5, 8]. These actively localizing transcripts are recognized by the transport machinery via cis-acting regions, also termed zip-code elements [9]. At the target site, mRNPs undergo reorganization and local protein synthesis is activated (Fig. **2A**) [6, 10]. Recent studies suggest that the individual steps of active transport are often closely connected and that, for instance, even co-transcriptional events influence the anchoring of mRNAs at their target site and their local translation. 

In this review, we will focus on the assembly of localized RNPs and their active transport to subcellular sites. In order to highlight general principles of mRNA localization, we will discuss three representative examples, for which a sufficient body of mechanistic insights is available. We will begin with summarizing the molecular events leading to the transport of *ASH1* mRNA in yeast. Studies on *ASH1* have let to the yet best characterization of an mRNA-localization mechanism. It is particularly well suited to exemplify the dynamics of mRNP assembly from the nucleus to its cytoplasmic destination. Since mRNA localization also serves functions that are specific to multicellular organisms, we will discuss two localization events occurring during the early development of the fruit fly [11, 12]*.* The first of these examples is the localization of *oskar* (*osk*) mRNA to the posterior pole of the *Drosophila* embryo. We chose this example because it demonstrates the requirements of nuclear splicing events as well as oligomerization of mRNAs for their localization. Such an involvement of RNA-centered steps may be important also in other organisms. The second example from *Drosophila* is the dynein-dependent localization of several mRNAs by a protein complex containing Egalitarian (Egl) and Bicaudal D (BicD). This example was chosen because it highlights the close collaboration of motor-associated components and RNA recognition factors during mRNA localization. It should be noted that mRNA localization is also important for many vertebrate oocytes and somatic cells, such as neurons and fibroblast. However, in this review we will not cover these localization processes because they have been subject of excellent recent reviews [7, 13-15]. 

## LOCALIZATION OF *ASH1* mRNA IN BUDDING YEAST

In *Saccharomyces cerevisiae*, a set of about 30 mRNAs are transported from the mother cell into the daughter cell at various stages of the cell cycle [16-19]. They accumulate at the tip of the bud or mature daughter cell, where they are supposedly translated [20-22]. The best-studied of these transcripts is *ASH1* mRNA. It encodes a transcriptional repressor that inhibits mating-type switching in the daughter cell and therefore ensures different cell fates of mother and daughter cell [23, 24]. About 15 years ago, the main factors required for *ASH1* mRNA localization were identified in a genetic screen [25]. Since then, a comprehensive understanding of the main steps of *ASH1*-mRNA localization has been obtained (Fig. **2B**) [20-22, 26]. 

Loading of RNA-binding proteins onto *ASH1* mRNA already begins in the nucleus with the binding of She2p, a key trans-acting factor for localization of *ASH1*. It has been reported that She2p interacts with transcriptionally active RNA polymerase II [27], suggesting that She2p can bind co-transcriptionally to zip-code elements of the nascent *ASH1* transcript. However, the specificity of this early aspect of pre-mRNP assembly is still being disputed [28] and requires further clarification. 

She2p has an unusual RNA-binding domain that consists of two dimers forming an elongated tetramer [29, 30]. Nucleic-acid binding is achieved by a large joint surface spanning both dimers and by two small helices protruding at each side from the tetramer [29, 30]. After transcription, She2p and *ASH1* mRNA pass through the nucleolus [31], where the nucleolar protein Loc1p binds to *ASH1* mRNA [32, 33] and She2p (Fig. **2B**) [34]. Although Loc1p does not leave the nucleus, its genomic deletion results in impaired cytoplasmic* ASH1* mRNA localization [33]. The mechanistic basis of Loc1p for *ASH1* mRNA localization has not yet been unraveled.

Two other nuclear shuttling factors, the RNA-binding proteins Puf6p and Khd1p, also interact with *ASH1* mRNA already in the nucleus (Fig. **2B**) [31, 34-36]. In contrast to Loc1p, they remain associated with *ASH1* mRNA during its cytoplasmic transport [35-38]. The function of both proteins is the translational repression of *ASH1* mRNA during its transport. 

The nuclear *ASH1* pre-mRNP is exported to the cytoplasm, where it associates with a cytoplasmic, motor-containing pre-complex [39]. It consists of the myosin adapter and RNA-binding protein She3p and its binding partner, the type V myosin Myo4p (Fig. **2B**) [40-46]. She3p directly binds to *ASH1* mRNA as well as to She2p and therefore has a central function in assembling the mature mRNP [28, 41, 45]. Whereas each of the two RNA-binding proteins alone has only moderate specificity for zip-code elements of localizing mRNAs, the ternary complex of She2p, She3p and localizing mRNAs shows strong synergistic binding with high specificity [28]. This specific recognition of localizing mRNAs by the maturing cytoplasmic complex constitutes an important quality control step for the transport of only the correct transcripts to the bud tip. 

With its C-terminal half She3p interacts with She2p to form the described specific ternary complex [28, 41, 45], whereas its association with Myo4p is mediated by She3p’s N-terminal half [40-44]. Together with cargo RNA, this minimal complex is able to exert directional movement [47]. In contrast to type V myosins from higher eukaryotes, Myo4p was reported to be non-processive [44, 48, 49] and monomeric in absence of binding partners [42, 44, 48]. Since dimerization has been reported to be important for processive movement of myosins [50], the question arose how transport along actin filaments can be achieved in the cell. Recent reports provided evidence that Myo4p is oligomerized in the mature cargo complex [47, 51]. These complexes undergo sustained movement [51], suggesting that activation of motility may be achieved by incorporation of multiple motors into the mRNP (Fig. **2B**). 

Translation of *ASH1* mRNA is controlled by the RNA-binding proteins Puf6p [36] and Khd1p [35]. Puf6p binds to the 3’ UTR of the *ASH1* mRNA [36] and simultaneously to the general translation factor eIF5B [37]. The latter interaction prevents assembly of 80S ribosomes on *ASH1* mRNA and its subsequent translation. Khd1p binds to the first half of the open reading frame of *ASH1* mRNA [35, 38]. Moreover, Khd1p interacts with the C-terminal domain of the general translation initiation factor eIF4G1 [38], most likely preventing the recruitment of the translation pre-initiation complex on *ASH1* mRNA. The combined action of Puf6p and Khd1p prevents premature translation of *ASH1* mRNA during transport. After reaching the site of destination the transport complex is anchored, disassembled and translation is activated. Whereas translation activation by release of the translational repressors is well understood [10, 21], anchoring and disassembly of the transport complex is mechanistically less explored. 

After transport, *ASH1* mRNA accumulates in a crescent-like shape at the plasma membrane, which has been interpreted as anchoring [39, 52, 53]. Mutations have been reported that abolish this crescent-like localization without impairing the transport *per se* [52]. Surprisingly, anchoring also appears to require active translation, as an *ASH1* mRNA with mutated start-codon shows strongly impaired crescent-like localization [35]. 

At the bud tip, translation is activated by phosphorylation of Khd1p and Puf6p. Khd1p becomes phosphorylated by the membrane-associated kinase Yck1p [38], which reduces its affinity for RNA and allows for cap-dependent translation initiation. Similarly, phosphorylation of Puf6p by casein kinase II results in a decrease of Puf6p affinity for *ASH1* mRNA. This allows assembly of the 60S subunit with the 40S on the AUG start codon and translational activation [37]. Such phosphorylation-dependent reduction of RNA affinity of transport core factors has also been described in vertebrates for the zip-code binding protein 1 (ZBP1) and its target, the β-actin mRNA [54]. Thus, phosphorylation-dependent activation of the translation of localizing mRNAs appears to be a conserved principle.

Besides *ASH1* mRNA more than 30 additional transcripts are transported by the She2p/She3p/Myo4p complex [16-19]. Like *ASH1*, they are recognized by the transport machinery via their cis-acting zip-code elements [41, 53, 55-58]. 

In yeast, the zip-code elements of localized mRNAs show only limited sequence or structural conservation and thus the features defining specificity have not been fully understood [41, 53, 55-58]. Several of these zip-code elements contain a stem-loop with bulged regions that are necessary for binding of She2p [59]. In a subset of these zip-code elements a sequence motif was identified to be required for localization [58]. This motif consists of a CGA base triplet in a loop and a single cytosine in a second loop, separated by a double-stranded RNA helix of 4-5 base-pairs in length. Because this motif is not fully conserved and it is not sufficient for binding [57], further work will be required to understand the properties that define SHE-dependent zip-code elements. 

Since all localized mRNAs in yeast are transported by the same machinery, the question arose whether each zip-code containing mRNA is localized independently or whether multiple transcripts are transported together in a large complex. Live-cell imaging combined with a dual tagging strategy of the localized mRNAs in fact showed that localizing mRNPs contain at least two different mRNAs [60]. This finding supports the notion that *ASH1* mRNPs might consist of larger structural assemblies that enable the co-transport of multiple transcripts to their destination.

## LOCALIZATION OF *OSKAR* mRNA IN THE EARLY *DROSOPHILA* EMBRYO

During *Drosophila* oogenesis, mRNA localization presents an initial key step for the establishment of the body axes and embryonic patterning. Several mRNAs of maternal origin, including *bicoid*, *gurken* and *osk*, are transported from the nurse cells into the oocyte and localized to distinct positions within the oocyte. After fertilization, their locally translated protein products provide positional information and establish a tightly controlled transcriptional regulatory network for the segmentation of the embryo [61]. The localization of mRNPs containing *bicoid* or *osk* has been studied in great detail [11, 62] and involves microtubule-dependent motor proteins such as kinesin and dynein [11]. Especially in the case of *osk*, its localization process can be structured into several phases, including mRNA export from the nucleus, dynein-dependent transport of *osk* from the nurse cells into the oocyte, and kinesin-dependent trafficking to the posterior pole of the oocyte. A number of RNA-binding proteins have been described that act in trans to facilitate the transport from the nurse cells into and within the oocyte [11]. Some of them also serve as translational repressor during the transport process.

Throughout its lifetime from synthesis in the nuclei of nurse cells to degradation at the posterior pole of the oocyte, the *osk* transcript is associated with a dynamic collection of proteins. These factors orchestrate the synthesis, processing, export, translational control, localization, and degradation of *osk* mRNA. More functionally relevant trans-acting factors are known for *osk* RNA than for any other localized transcript.

During splicing in multicellular eukaryotes, a large multisubunit complex called the exon junction complex (EJC) is deposited upstream the exon-exon junction. Whereas this complex generally serves as a hallmark for the nonsense-mediated decay of mRNAs with premature stop codons [63], its assembly upstream of the first exon-exon junction is essential for *osk* mRNA localization [64]. This requirement nicely fits genetic data showing that *osk* reporter mRNAs derived from cDNA are incompetent of localization in the absence of endogenous *osk* mRNA. In addition to EJC components, several other RNA-binding proteins whose loss of function result in defects in *osk* localization, shuttle between nucleus and cytoplasm. These include proteins of the heterologous nuclear RNP (hnRNP) family such as Hrp48 and Squid/Hrp40. Whereas Hrp48 directly binds to *osk* 5’- and 3’-UTR [65, 66], Hrp40 interacts only with *osk* 3’-UTR where it also binds to Hrp48 [67]. Since many hnRNP proteins bind RNA co-transcriptionally [68], Hrp40 and Hrp48 likely also assemble with *osk* in the nucleus. Unlike other hnRNPs binding to *osk*, the polypyrimidine tract-binding protein (PTB)/hnRNP I does not need to bind its target mRNA inside the nucleus. This conclusion was drawn based on the observation that an exclusively cytoplasmic variant of PTB is able to associate with *osk* and functionally replace endogenous PTB [69]. In contrast to the *Drosophila* protein, the nuclear association of the *Xenopus laevis* PTB homolog with its target *Vg1* mRNA has been proposed to be a crucial step during localization [70]. *Drosophila* PTB binds to multiple sites within the *osk* 3’-UTR and mediates the formation of large complexes containing multiple *osk* molecules [69]. This assembly might serve at least two functions, packaging multiple mRNA molecules into mRNPs for efficient transport and repression of *osk* translation by masking the mRNA from the translation machinery. 

Interestingly, formation of large *osk* RNA protein particles also involves a second translational repressor, Bruno [71]. Bruno contains three RNA-Recognition Motifs (RRM), binds to several sites within *osk* 3’-UTR (Bruno response elements, BRE) and appears to repress translation via two different mechanisms. On one hand, Bruno recruits Cup, an inhibitor of cap-dependent translation initiation that interferes with the interaction of the translation initiation factors eIF4E and eIF4G [72]. On the other hand, *in vitro* observations suggest that by binding to its cognate sites within *osk* mRNA the protein incorporates the transcripts into large 50 - 80S translation silencing particles [73]. This packaging of mRNA renders it inaccessible for the translation apparatus. Similar to Bruno, Hrp48 binds to BREs [66]. Life imaging of *osk* RNP particles has recently revealed that Hrp48 is also required for formation of large *osk* particles [74]. Additional evidence for a function of BRE in multimerization of *osk* mRNA comes from observations that BRE elements can act in trans and establish translational control on co-expressed *osk* mRNA mutants that lack BREs [75]. This finding is consistent with the idea that *osk* RNP particles contain multiple *osk* RNA molecules with their corresponding RNA-binding proteins. Besides protein-driven multimerization, new data also suggest that RNA-RNA interaction between individual *osk* molecules could contribute to the formation of these large particles. A stem-loop region within *osk* 3’-UTR that does not encompass any known binding site for the above mentioned proteins is sufficient to drive homodimerization of two *osk* messages [76]. Together these data suggest that protein- as well as RNA-mediated formation of large particles is crucial, both for translational repression and transport of *osk* mRNA. 

The large *osk *particles described above contain additional RNA binding proteins like Exuperantia (Exu) and Staufen (Stau). Exu lacks canonical RNA binding motifs but associates with *osk* mRNA and with many proteins involved in translational repression of *osk* [77]. Exu is required for proper *osk* mRNA localization and found in RNPs that display dynamic movements consistent with active transport. Staufen contains several double-stranded RNA binding domains (dsRBDs) and is involved in RNA localization in a number of organisms [78]. On one hand, genetic data provide evidence that it is involved in anchoring at the end of transport. On the other hand, Stau is a component of the large *osk* mRNPs already early on during microtubule-dependent transport [79]. Although in mammalian cells, at least a subfraction of both Staufen homologs, Stau1 and Stau2, shuttle between nucleus and cytoplasm [80], it has been demonstrated that XStau, the *Xenopus* homolog that participates in localization of *Vg1* mRNA in oocytes, assembles with the RNA in the cytoplasm after nuclear export [70]. Similarly, *Drosophila* Stau associates with the mature *osk* particle in the cytoplasm, presumably after the transport of *osk* particles from the nurse cell to the oocyte [74]. In the oocyte, *osk*-containing mRNPs are localized to the posterior pole via active transport by microtubule-dependent motor proteins [74, 81]. The transport is at least in part mediated by the plus end directed motor kinesin-1. Interestingly, tracking of *osk* mRNPs in living oocytes has revealed that this transport corresponds to a random walk [81] and might actually reflect directed, motor-dependent movement along a weakly polarized microtubule network [82]. Transport is followed by anchoring or local entrapment at the oocyte’s posterior pole. This entrapment depends on components of the actomyosin system [83] but also on RNA binding proteins like Staufen. 

In summary, the detailed analysis of *osk* mRNA localization has revealed that also in multicellular organisms multiple RNA-binding proteins participate in the localization of an mRNA and that nuclear events are important to guide cytoplasmic localization of transcripts. Some of the described proteins like Stau, Hrp48, or EJC components such as Barentz might have a more direct role in mRNA transport by e.g. recruiting different motor proteins (dynein or kinesin I) at various stages of localization. The involved RNA-binding proteins can have diverse but also overlapping functions during localization, ranging from translational control to particle formation and anchoring at the target site.

## MINUS-END DIRECTED LOCALIZATION OF TRANSCRIPTS EARLY IN *DROSOPHILA* DEVELOPMENT

During *Drosophila* development, directional transport of several mRNAs also occurs in a dynein-dependent manner towards the minus-ends of microtubules. Cytoplasmic dyneins are unrelated to myosins or kinesins and much larger [84]. The functional, dynein-containing motor complex consists of several heavy and light chains. The dynein heavy chains form a central ring mainly consisting of six AAA ATPase-related domains [85], of which two are tethered together in larger complexes. Light chains and adapters associate with this core structure, regulate the motor function and mediate its binding to several cargos [86]. Of these dynein interactors, the multiprotein dynactin complex is of particular importance. It is required for the motor binding to several cargos and modulates motor processivity [86]. 

During *Drosophila* oogenesis, dynein motors transport several transcripts from the nurse cells into the oocyte. Later in the blastoderm embryo this motor complex also localizes mRNAs to its apical periphery [11, 87]. In both cases, transport occurs towards the minus-ends of microtubules. Dynein-dependent cargo mRNAs include *bicoid***, ***fushi tarazu*, *gurken*, *hairy*, *fs(1) K10* (*K10*), *orb***, ***wingless*, and the *I Factor* retrotransposon RNA [88-94].

For *osk* mRNA as well as for *ASH1* mRNA a nuclear history is important for their respective cytoplasmic localization. For dynein-dependent transport during *Drosophila* development it remains unclear whether specific nuclear events are also required. Microinjection of dynein-dependent transcripts into *Drosophila* embryos resulted in their efficient localization to the apical periphery [94]. Although it is difficult to judge from these experiments whether the transport occurs with full efficiency, it rather supports the notion that a nuclear history for mRNA transport may not always be required.

During transport of mRNAs to apical sites of the *Drosophila* embryo, trafficking mRNPs switch their movement frequently from the minus-end direction towards the apical periphery to plus-end direction and vice versa [95, 96]. In contrast to the minus-end directed, dynein-dependent movement of these mRNPs [94], the molecular motor(s) for basal transport towards plus ends has not been unambiguously identified. However, it has been suggested that plus-end directed movement might be achieved by the modulation of dynein-dynactin complexes [96, 97]. A rather surprising finding was that even non-localizing mRNAs are subject to active transport [95]. It indicates that the specificity of the involved RNA-binding proteins might not be very high. Moreover, the observed unspecific transport was almost exclusively bidirectional. In contrast, apically localized mRNAs showed an increased probability to initiate and maintain fast minus-end directed movement [95].

One puzzling feature of zip-code elements that mediate minus-end directed transport in *Drosophila* embryos is the lack of conserved sequence motifs [59]. Recently, the three-dimensional structure of a 44-nt long zip-code element of the *Drosophila* *K10* mRNA [92], which is transported from the nurse cells into the oocyte [98], was determined by nuclear-magnetic resonance (NMR) spectroscopy [99]. The study revealed that purine-base stacking within the double-stranded stem-loop of this zip-code element leads to a distortion of the helix, a widened major groove, and the local formation of a so-called A'-form helix. Injection of *K10* zip-code RNA into blastoderm embryos efficiently localized to apical sites, whereas mutant versions with a narrower major groove showed impaired apical localization [99]. This example demonstrates the close links between sequences and structural features. Because endogenous *K10* mRNA is expressed and transported much earlier in development than the developmental time-point of the injection experiments [98], it can be concluded that the special properties identified in the *K10* zip-code element are likely to be general features of zip-code elements for minus-end directed dynein transport.

The similarity of these processes in oogenesis and embryogenesis also extends to the involved protein co-factors. During both developmental stages, the two proteins Egalitarian (Egl) and Bicaudal D (BicD) are expressed and required for minus-end localization of transcripts [100, 101]. Egl contains a domain with similarity to 3'-5' exonucleases and binds RNA through an unidentified domain, whereas BicD is a dynein cofactor without apparent RNA-binding features [102]. Both proteins form a co-complex that associates with dynein light chain and recruits the dynein/dynactin complex [101-104]. 

The RNA-binding properties of Egl suggested that it mediates the recognition of zip-code RNAs. Alone, Egl showed a modest preference for zip-code containing RNAs over non-localizing control RNAs [102]. The presence of BicD resulted in the formation of a ternary complex with Egl and RNA, and further increased the preference for localizing transcripts over a mutated zip-code RNA. Because BicD does not bind directly to RNAs, this protein might have a positive allosteric effect on Egl's binding to zip-code RNAs. In summary, the selectivity of the dynein-associated transport machinery for zip-code containing RNAs seems not very high *in vitro*. This is consistent with the observed bidirectional, unbiased transport of non-localizing mRNAs *in vivo* [95]. However since apical transport of mRNAs is an efficient process, it seems rather likely that in the embryo specificity is higher and might even require an additional co-factor of the Egl-BicD complex.

An obvious question is how the recognition of zip-code mRNAs by Egl induces a net bias of movement towards minus ends of microtubules, whereas non-localizing RNAs are transported bidirectionally. The RNA-Egl-BicD complex might stabilize the dynein complexes and thereby increases the total copy number of active dynein motors associated with localizing transcripts. This hypothesis receives support from *in vivo* injection experiments [95]. It has also been suggested that the modulation of higher order properties of motor complexes instead of shear copy numbers might be able to dictate directionality of cargo transport [105]. Alternatively, the complex formation could alter the motile activity of either the dynein motor or its plus-end directed antagonist. It seems obvious that the clarification of this and related issues will require further investigation.

Several different transcripts are recognized and localized to apical sites of the embryo. In principle, they could either be transported in distinct particles or be co-transported in joint assemblies. Co-injection experiments with different fluorescently labeled, zip-code containing RNAs demonstrated that they are indeed incorporated into the same particles and transported together to apical sites [94]. No such co-localization was observed for non-localizing RNAs, again suggesting high specificity of cargo selection for their transport. 

At the minus-end destination of dynein transport, the mRNA becomes anchored. For the apical attachment of transcripts, the dynein motor becomes a static tethering factor [106]. Antibody injection experiments further suggested that also the local accumulation of *gurken* mRNA in the oocyte after its dorso-anterior transport depends on dynein as a static tethering factor [107]. Although Egl and BicD are required for the transport of *gurken* mRNA, they are dispensable for its anchoring. Together these findings suggest that dynein-dependent transport and anchoring of mRNAs follow similar principles in the oocyte and in the blastoderm embryo. It will be interesting to see whether this assumption holds true also for minus-end directed transport in adult tissues and in higher eukaryotes.

## GENERAL PRINCIPLES AND PERSPECTIVES

The direct comparison of the presented mRNA localization events suggests that in multicellular organisms rapidly alternating, bidirectional transport events by antagonizing motors is a common feature. In contrast in lower species like yeast either only one type of motor is involved or the opposing motors do not seem to constantly change direction of the transported particles. Although too few examples have been studied in sufficient detail to fully support this hypothesis, the general trend seems to follow this rule. In *S. cerevisiae* SHE-dependent RNA transport and Myo2p-dependent vesicle and organelle transport both involve only a single type V myosin motor [50]. In the filamentous fungus *Ustilago maydis* hyphal transport of dozens of mRNAs also depends on directional transport [3]. Although these RNAs are shuttled bidirectionally along microtubules, particles do not constantly change their direction [108] and thus appear to follow principles more similar to mRNA localization in *S. cerevisiae* than in corresponding bidirectional events in *Drosophila*. As switching of direction is also a common feature in neurons [109-113], bidirectional transport indeed seems to be more frequent in higher eukaryotes. This observation is also consistent with the frequent finding of more than one type of motor proteins in such particles. Furthermore, in lower species myosin-dependent transport functions exclusively towards the plus-end of actin, whereas for metazoan also minus-end directed myosins have been described. In future it will be necessary to address questions in higher eukaryotes that are similar to the ones currently studied in *Drosophila*. A major drawback, however, is the enormous complexity of mRNA-localization particles for instance in neurons [13, 114]. Thus, for the coming years less complex model system like yeast and *Drosophila* that can be genetically challenged will continue to guide the way to a mechanistic understanding of mRNA-localization events in general. 

Yet another open issue is how specific individual mRNA transport events really are. A major problem with many mRNA localization events is the lack of biochemical insights into the composition and assembly of their corresponding mRNPs by reconstitution experiments where binding affinities can be quantified under controlled conditions. To date only in *S. cerevisiae* such reconstitution experiments yielded high mRNA specificities that are sufficient to explain specific transport *in vivo* [28]. In the case of apical localization of mRNAs by the Egl-BicD containing dynein complex in *Drosophila* a preference has been reported for zip-code RNAs [102]. However, no highly specific binding was observed. It suggests that either additional co-factors are necessary to achieve full specificity or that folding or posttranslational modifications of participating factors only occur correctly in its endogenous environment. 

An example where high specificity for RNA binding may not be required is mRNA localization in the fungus *U. maydis*. Here, a range of RNAs are transported back and forth along microtubules without anchoring at a target site [3]. It seems likely that the RNAs are not transported to achieve tight temporal and spatial control over expression of the encoded proteins but rather to ensure even distribution of a range of mRNAs throughout the extremely elongated interior of the cell. Thus, depending on the respective functions, cargo specificities may vary substantially. 

When comparing the reported *in vitro* specificities of RNA-binding proteins with their apparent function *in vivo*, an interesting discrepancy can be observed. On one hand, several isolated RNA-binding domains lack high specificity *in vitro*, whereas in their cellular context their full-length proteins are involved in highly specific recognition events [2, 115]. This apparent discrepancy could be explained by the observation that in several cases RNA-binding domains cooperatively interact with target RNAs to ensure a more selective binding. For instance, the proteins Sex-lethal, Hrp1, and HuD each uses two RRMs to cooperatively recognize their target RNAs [116-118]. Other examples include the above-described cooperative *ASH1* mRNA binding by the two transacting factors She2p and She3p [28] and the recently published zip-code RNA binding by two KH-domains of ZBP1 via intramolecular cooperativity [119]. Because many RNA-binding proteins in higher eukaryotes contain multiple RNA-binding motifs [115], it is tempting to speculate that such cooperativity for specific RNA binding is a very general feature of mRNA localization. 

## NOTE ADDED IN PROOF

The following studies have been published after acceptance of this manuscript and should be considered for further reading:

- Ghosh S., Marchand V., Gáspár I., Ephrussi A., Control of RNP motility and localization by a splicing-dependent structure in oskar mRNA. *Nat Struct Mol Biol* 2012, *19 *(4), 441-9.
- Amrute-Nayak M., Bullock S.L., Single-molecule assays reveal that RNA localization signals regulate dynein-dynactin copy number on individual transcript cargoes. *Nat Cell Biol* 2012, *14*(4), 416-23.
